# Gene Expression Signatures That Predict Radiation Exposure in Mice and Humans

**DOI:** 10.1371/journal.pmed.0040106

**Published:** 2007-04-03

**Authors:** Holly K Dressman, Garrett G Muramoto, Nelson J Chao, Sarah Meadows, Dawn Marshall, Geoffrey S Ginsburg, Joseph R Nevins, John P Chute

**Affiliations:** 1 Institute for Genome Sciences and Policy, Duke University Medical Center, Durham, North Carolina, United States of America; 2 Department of Molecular Genetics and Microbiology, Duke University Medical Center, Durham, North Carolina, United States of America; 3 Division of Cellular Therapy, Department of Medicine, Duke University Medical Center, Durham, North Carolina, United States of America; Fred Hutchinson Cancer Research Center, United States of America

## Abstract

**Background:**

The capacity to assess environmental inputs to biological phenotypes is limited by methods that can accurately and quantitatively measure these contributions. One such example can be seen in the context of exposure to ionizing radiation.

**Methods and Findings:**

We have made use of gene expression analysis of peripheral blood (PB) mononuclear cells to develop expression profiles that accurately reflect prior radiation exposure. We demonstrate that expression profiles can be developed that not only predict radiation exposure in mice but also distinguish the level of radiation exposure, ranging from 50 cGy to 1,000 cGy. Likewise, a molecular signature of radiation response developed solely from irradiated human patient samples can predict and distinguish irradiated human PB samples from nonirradiated samples with an accuracy of 90%, sensitivity of 85%, and specificity of 94%. We further demonstrate that a radiation profile developed in the mouse can correctly distinguish PB samples from irradiated and nonirradiated human patients with an accuracy of 77%, sensitivity of 82%, and specificity of 75%. Taken together, these data demonstrate that molecular profiles can be generated that are highly predictive of different levels of radiation exposure in mice and humans.

**Conclusions:**

We suggest that this approach, with additional refinement, could provide a method to assess the effects of various environmental inputs into biological phenotypes as well as providing a more practical application of a rapid molecular screening test for the diagnosis of radiation exposure.

## Abstract 2

John Chute and colleagues report that gene expression patterns in peripheral blood mononuclear cells from mice and humans reflect prior radiation exposure.

## Editors' Summary

**Background.:**

Everyone living on earth is constantly exposed to low levels of ionizing radiation—energy in the form of waves or particles that is powerful enough to strip electrons out of atoms and to break chemical bonds in important biomolecules. These low levels of ionizing radiation come from radioactive chemicals in the ground and cosmic rays, for example, and are relatively harmless. Occasionally, though, individuals are exposed to larger amounts of ionizing radiation, often as a result of medical tests and treatments but sometimes through the accidental or deliberate release of radioactive chemicals. These larger doses, which permanently damage or kill cells, can cause radiation sickness, a condition characterized by bone marrow failure, gut problems, susceptibility to bacterial infections, and other symptoms that develop days or months after exposure to ionizing radiation. Particularly large doses can be lethal but even moderate doses can increase an individual's risk of developing cancer later in life.

**Why Was This Study Done?:**

Some of the effects of ionizing radiation can be reduced if suitable treatment is started immediately after exposure. Unfortunately, it takes several days to estimate the amount of ionizing radiation to which an individual has been exposed. It would be useful to measure personal exposures more quickly, especially in emergency situations where ideally doctors would be able to distinguish rapidly and accurately between the “worried well” and exposed individuals. As cells respond to irradiation by altering the expression of some genes, the researchers in this study investigated whether gene expression profiling (a molecular biology technique that catalogues all the genes expressed by a cell) can be used to define a set of gene expression changes—called a metagene—that differentiates between irradiated and non-irradiated cells.

**What Did the Researchers Do and Find?:**

The researchers exposed mice to no ionizing radiation, a low dose that causes no medical problems, an intermediate dose that damages blood cells, or a lethal dose. Six hours later, they isolated blood cells from the mice, and catalogued which genes each sample expressed. Using this information, the researchers identified and validated metagenes that accurately distinguished between blood samples from non-irradiated and irradiated animals and between samples from animals exposed to different radiation doses. The researchers then developed a metagene for human radiation exposure using blood samples taken from patients before and after total body irradiation given as part of their medical treatment. This metagene correctly identified 18 of 20 pre-irradiation samples and 17 of 20 post-irradiation samples. Finally, the researchers tested whether the radiation metagenes developed in mice could distinguish between samples taken from irradiated and non-irradiated people. Although the high-dose mouse metagene correctly identified all of the samples from healthy donors as being non-irradiated, it correctly identified only two-thirds of the pre-irradiated samples from patients.

**What Do These Findings Mean?:**

These findings indicate that metagenes can be generated that recognize different levels of radiation exposure in mice and people. In the mouse study a metagene was identified that correctly identified in all cases whether a sample came from a non-irradiated mouse or an animal exposed to the lowest dose of radiation. This result suggests that it might be possible to use a metagene to identify exposed individuals among thousands of “worried well” after a radiation emergency. First, however, the mouse and human metagenes identified here need to be refined to improve their accuracy and then validated in more people. The current high-dose mouse metagene may be bad at identifying non-irradiated patients, for example, because of gene expression changes that are a result of the patients' underlying disease or previous medical treatments. By studying additional patients, it might be possible to improve the accuracy of the metagene by taking these radiation-independent changes into account. Finally and more generally, these findings suggest that the metagene approach could be used to monitor people's exposure to other dangerous environmental agents.

**Additional Information.:**

Please access these Web sites via the online version of this summary at http://dx.doi.org/10.1371/journal.pmed.0040106.

- US Environmental Protection Agency offers information on understanding radiation and factsheets on ionizing radiation
- MedlinePlus provides links to information on radiation exposure and pages on radiation sickness
- US Centers for Disease Control and Prevention has information on emergency preparedness and response to radiation emergencies
- Wikipedia has pages on ionizing radiation, radiation poisoning, and expression profiling (note that Wikipedia is a free online encyclopedia that anyone can edit)

## Introduction

Environmental risks and individual genetic repertoires are considered to be the primary influences that dictate a person's susceptibility to disease. However, for any given disease, accurate estimation of the contribution of either environmental influences or genotype to disease development can be difficult. Once an association between a given environmental exposure and a particular biological phenotype has been suggested, it is important, if possible, to prospectively determine whether the environmental exposure has a causative or predictive impact on the biological phenotype.

Ionizing radiation is an example of a ubiquitous and potentially dangerous environmental hazard that has been associated with the development of thyroid cancers, multiple myeloma, and myeloid leukemias in children and adults [1–3]. Following the atomic bombings at Hiroshima and Nagasaki, many studies were performed to assess the incidence of specific genetic mutations, such as *p53* and *HPRT* mutations, in somatic cells from survivors of these events [4–6]. Furthermore, Neel et al. performed comprehensive mortality and phenotype studies of children of atomic bomb survivors, which surprisingly have not revealed an overt increase in mutagenesis or teratogenesis in F_1_ offspring [7–9]. However, a comprehensive characterization of the genetic changes that can occur in human populations exposed to ionizing radiation (e.g., Chernobyl reactor accident victims) has not been performed, and quantification, at the genetic level, of the impact of radiation exposure on the risk of developing such diseases has not been measured.

Recently, the potential hazard of ionizing radiation exposure has been identified as both a public health and national security risk [10–13] in light of the anticipated use of radiological or nuclear materials by terrorists to make “dirty bombs” or “improvised nuclear devices” [10–13]. In addition, preliminary studies have now been performed utilizing gene expression analysis of tumor cells and cell lines [14,15], primary cells [16], rodents [17], and peripheral blood (PB) from small numbers of patients [18] to identify genes whose expression is altered following exposure to radiation. In the event of a dirty bomb or a higher-impact nuclear detonation, thousands of individuals may present for immediate medical attention to determine whether they have been exposed. It would be critical in such an event for caregivers to have the capability to rapidly triage which individuals have received deterministic exposures versus the “worried well”; biological dosimetry becomes even more critical when considered in light of the limitations of the current tools available to estimate an individual's exposure level. Lymphocyte depletion kinetics require several (>3–7) daily complete blood counts to provide accurate prediction of dose received, and decline can lag for 48 hours even in heavily exposed individuals [12,13]. Cytogenetics analyses are the current gold standard to measure the dicentric DNA breaks that occur following radiation exposure [12,13,19] but require several days to complete.

One approach to achieve a faster and potentially highly sensitive measurement of radiation exposure would be the utilization of high-throughput gene expression analyses to identify patterns of molecular changes that occur following exposure. Such an approach, targeting a radiosensitive and easily accessible cell population, such as PB lymphocytes [20], could potentially lead to a validated panel of “radiation response” genes that have yet to be identified and could be translated into a rapidly applicable diagnostic screening test. Our group has utilized genomic analyses to identify genes predictive of prognosis within several types of cancers [21–24] as well as genes that predict patient response to chemotherapy [21]. Gene expression analysis of PB leukocytes has also been applied to distinguish patients with atherosclerosis from individuals without it [25] and to identify variations in gene expression among healthy individuals [26]. We sought to determine whether a similar strategy could be applied to determine which genes will predict different levels of radiation exposure and possibly allow stratification of individuals on the basis of their genomic profiles. We have demonstrated that genome-scale measures of gene expression, together with advanced computational tools, can successfully generate molecular signatures that distinguish clinically relevant levels of radiation exposure in mice and humans.

## Methods

### C57B16 Murine Irradiation Studies

We housed ten-week-old C57Bl6 female mice (Jackson Laboratory, http://www.jax.org) at the Duke Cancer Center Isolation Facility and studied them under specifications approved by the Duke University Animal Care and Use Committee. Mice (*n* = 7–10 per group) were irradiated with either 50 cGy total body irradiation (TBI), 200 cGy, or 1,000 cGy delivered by a Cs137 irradiator at a dose rate of 480 cGy/min. PB (500 μl) was collected via ocular bleed from each irradiated mouse 6 h following exposure of the mice to TBI, and an equal amount was collected from nonirradiated control mice. PB mononuclear cells (MNCs) were collected via Ficoll-Hypaque centrifugation, and total RNA was isolated from these cells as we have previously described [27]. Total RNA quality was assessed by an Agilent Bioanalyzer 2100 (Agilent Technologies, http://www.agilent.com).

### Human Irradiation Studies

Patients undergoing TBI as part of their pretransplantation conditioning and healthy donors were enrolled to participate in this study following a protocol to collect PB samples that was previously approved by the Duke University Institutional Review Board. All patients receiving nonmyeloablative conditioning were treated with 200 cGy of TBI from a linear accelerator at a dose rate of 20 cGy/min. All patients who underwent TBI-based myeloablative allogeneic or autologous stem-cell transplantation received radiation fractionated at 150 cGy per fraction at 20 cGy/min. All patients had PB collected (50 ml) prior to and 6 h following exposure to either 200 cGy or 150 cGy radiation treatment. Certain patients also received 30 mg/m^2^ of fludarabine intravenously on days −5 through −2 and 500 mg/m^2^ of cyclophosphamide intravenously on days −5 through −2 as further immunosuppressive therapy. The irradiation was administered one day prior to the initiation of the fludarabine and cyclophosphamide, and the PB samples were drawn prior to the exposure to these immunosuppressive agents. MNCs were collected from each patient's PB using the identical Ficoll Hypaque methodology described above for mouse MNC collection, and total RNA was isolated.

### DNA Microarrays

Mouse and human oligonucletotide arrays were printed at the Duke Microarray Facility using Operon's Mouse Genome Oligo set (version 3.0) (https://www.operon.com), which contains 31,769 70-mer probes representing 24,878 genes and 32,829 transcripts, and the Operon Human Genome Oligo set version 3.0, which possesses 34,580 optimized 70-mers, representing 24,650 genes.

### RNA and Microarray Probe Preparation and Hybridization

Briefly, 5 × 10^6^ MNCs were pelleted, and total RNA was isolated using the RNAeasy minispin column [27]. Total RNA (2 μg) from each sample (mouse or human) and the universal reference RNA (Universal Human or Mouse Reference RNA, Stratagene, http://www.stratagene.com) were used in probe preparation. The reference RNA allows for the signal for each gene to be normalized to its own unique factor allowing comparisons of gene expression across multiple samples. This serves as a normalization control for two-colored microarrays and an internal standardization for the arrays. The relative ranges of gene expression for each analysis were measured by using the median of ratios (sample/Stratagene universal reference). Briefly, reverse transcription was driven by an oligo (dT) primer bearing a T7 promoter using ArrayScript. The cDNA then underwent second-strand synthesis and clean-up to become a template for in vitro transcription with T7 RNA polymerase. To maximize RNA yield, Ambion's (http://www.ambion.com) proprietary MEGAscript in vitro transcription (IVT) technology was used to generate amplified RNA (aRNA). The antisense aRNA was then fluorescently labeled with Cy3 (reference) and Cy5 (sample). Sample and reference aRNAs were pooled, mixed with 1× hybridization buffer (50% formamide, 5× SSC, and 0.1% SDS), COT-1 DNA, and poly-dA to limit nonspecific binding, and heated to 95 °C for 2 min. This mixture was pipetted onto a microarray slide, a cover slip was placed, and it was hybridized overnight at 42 °C. The array was then washed at increasing stringencies, and scanned on a GenePix 4000B microarray scanner (Axon Instruments, http://www.axon.com). Detailed protocols are available on the Duke Microarray Facility Web site (http://microarray.genome.duke.edu/). The expression levels of representative genes from the gene array analysis were confirmed via real-time quantitative RT-PCR analysis. The High Capacity cDNA Archive kit (Applied Biosystems, http://www.appliedbiosystems.com) was used to generate cDNA from total RNA from each sample. Each sample was assayed in duplicate using 5-ng cDNA (TaqMan Mastermix and TaqMan Gene Expression Assays real-time PCR primers ([Applied Biosystems]) and analyzed on an ABI Prism 7900HT Sequence Detection system (Applied Biosystems) according to the manufacturer's protocol.

### Data Processing and Statistical Analysis

Genespring 6.1 (Agilent Technologies) was used to perform initial data filtering in which spots whose signal intensities below 100 in either the Cy3 or Cy5 channel were removed. To then account for missing values, PAM software (http://www-stat.stanford.edu/∼tibs/PAM/) was used to impute missing values. *k*-nearest neighbor was used where missing values are imputed using a *k*-nearest neighbor average in gene space. For the hierarchical clustering analysis only those genes that varied across all conditions (2,213 probes) were applied through GeneCluster 3.0 [28]. Genes and samples were clustered using average linkage with centered correlation similarity metric, and the results were visualized in JavaTreeview [29]. Gene expression data in the mouse and human predictive analyses were filtered to exclude probe sets that had signal intensities below background signal level as well as genes that did not vary significantly across samples (6,793 mouse probes and 11,319 human probes). The expression signature for each dose response represents a group of genes as a single expression profile and is here derived as the top principal component, or metagene. Prediction analysis of the expression data was performed using MATLAB software as previously described for the analysis of breast cancer samples [22]. When predicting levels of radiation exposure, gene selection and identification is based on training the data and finding those genes most highly correlated to response. Each signature summarizes its constituent genes as a single expression profile and is here derived as the first principal component of that set of genes (the factor corresponding to the largest singular value), as determined by a singular value decomposition. Given a training set of expression vectors (of values across metagenes) representing two biological states (nonirradiated and irradiated), a binary probit regression model is estimated using Bayesian methods. Bayesian fitting of binary probit regression models to the training data then permits an assessment of the relevance of the metagene signatures in within-sample classification, and estimation and uncertainty assessments for the binary regression weights mapping metagenes to probabilities of radiation exposure. To internally validate the predictive capacity of the metagene profiles, we performed leave-one-out cross validation studies as we have previously described [22]. To externally validate the mouse-metagene profiles, we validated against prospectively collected, blinded human PB samples. To map the probe sets across species (mouse to human) we used an in-house program Chip Comparer (http://tenero.duhs.duke.edu/genearray/perl/chip/chipcomparer.pl). Each Operon probeset ID was mapped to a corresponding LocusID. This mapping was done by parsing local copies of LocusLink and UniGene databases to identify the inherent relationship between the GenBank accession number associated with each probeset sequence and its corresponding LocusID. Probesets from different arrays are matched by sharing the same orthologous pair of LocusIDs (across species). A total of 7,101 probes was mapped between the mouse and human operon probe sets and was used in validating the mouse model in the human samples. These patterns were then applied against the human samples in a blinded manner to determine the predictive capacity of each metagene profile against human PB samples from nonirradiated and irradiated patients. Genes found to be predictive of radiation dose were characterized utilizing an in-house program, GATHER (http://meddb01.duhs.duke.edu/gather/). GATHER quantifies the evidence supporting the association between a gene group and an annotation using a Bayes factor [30]. All raw data files are found at http://data.cgt.duke.edu/Chute.php.

## Results

### Development of a Radiation-Response Gene Expression Signature

**Figure 1:**
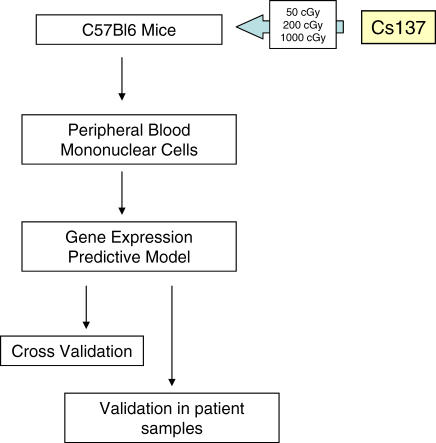
Scheme for Development of Radiation Response Expression Signature PB MNCs were collected from control (0 cGy), 50 cGy-irradiated, 200 cGy-irradiated, and 1,000 cGy-irradiated C57Bl6 mice. Gene array analyses were performed on RNA isolated from *n* = 7 replicates from each condition, and metagene profiles were developed to represent the four different levels of radiation exposure. A leave-one-out cross-validation study was performed, which revealed the highly predictive nature of these metagene profiles. Finally, these profiles were independently validated by blinded analysis of PB samples from human patients who had undergone TBI to determine the capacity for these profiles to predict human radiation exposure.

**Figure 2:**
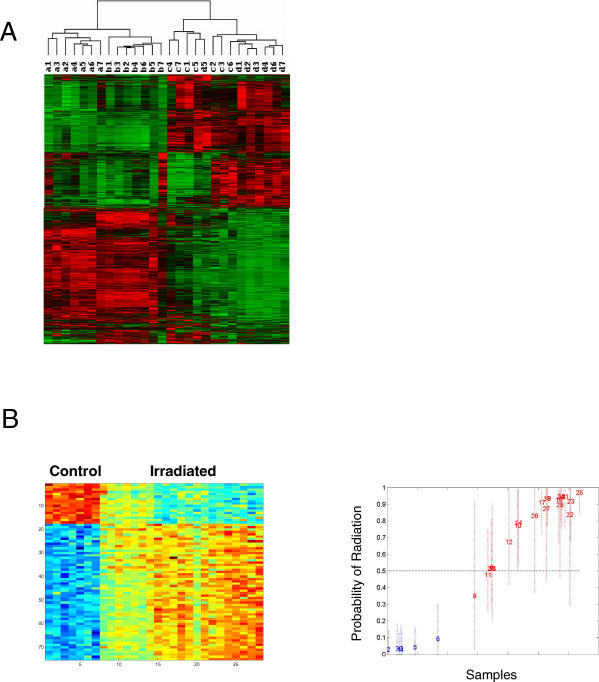
Gene Expression Profiles that Reflect Radiation Exposure (A) Clustering of samples based on gene expression patterns reflects radiation exposure. Hierarchical cluster analysis of a filtered list of 2,213 probe sets from expression data of PB MNCs from mice irradiated at varying dosages is shown (a, normal; b, 50 cGy; c, 200 cGy; d, 1,000 cGy). Each gene is represented by a single row, and each sample is represented by a single column. The color red represents expression ratios of overexpressed genes, green represents expression ratios of underexpressed genes, and black represents expression ratio of 0 (similar expression in both samples). The relative expression ranges from 0.02 to 610. (B) A supervised analysis to identify an expression profile that distinguishes control samples from irradiated samples is shown. On the left, PB MNCs were prepared from either control mice or mice irradiated at the indicated dose. RNA was extracted and used for the synthesis of probes for microarray analysis. The left images depict the expression pattern of genes selected for classifying irradiated samples from control. The expression of genes is standardized to zero mean and unit variance across samples, which are displayed with each row as a gene ordered vertically by the estimated regression weights. Each column is a sample from an independent experiment. High expression is depicted as red, and low expression is depicted as blue, and the range of expression is from 0.02 to 610. The images on the right depict leave-one-out cross validation of the classification probabilities at each dose. Each sample, including the controls and the irradiated samples, is predicted as a probability of exhibiting a gene expression signature reflecting the irradiated sample's specific pattern along with 95% confidence intervals indicated as dashed vertical lines. Each sample is plotted as its predicted probability of radiation response (red) versus nonresponse (blue) on the basis of the analysis of the remaining samples. Each sample is represented as a number in the chronological order of the dose of radiation received; e.g., samples 1–7 are from nonirradiated controls, 8–14 are from 50-cGy irradiated animals, 15–21 are from 200-cGy irradiated animals, and 22–28 are from 100-cGy irradiated animals.

As a strategy to develop gene expression profiles that could predict different levels of radiation exposure, ten-week-old C57Bl6 mice (*n* = 7 per group) were exposed to either 50 cGy, 200 cGy, or 1,000 cGy TBI as a single fraction from a Cs137 gamma source at a dose rate of 480 cGy/min (Figure 1). We chose these doses of irradiation since each represents an exposure with different expected medical implications. For example, 50 cGy exposure is low level and causes no acute medical deficits; 200 cGy is both immunosuppressive and myelosuppressive and could cause important clinical sequelae (e.g., infections) requiring medical intervention (i.e., antibiotics and growth factor administration); 1,000 cGy would likely be a lethal exposure despite maximal supportive care (i.e., transfusion support and antibiotics). We compared mice irradiated at these doses with nonirradiated control mice (0 cGy). PB samples (500 μl) were collected from each mouse 6 h following irradiation, PB MNCs were isolated via Ficoll Hypaque centrifugation, and total RNA was extracted from the PB MNCs from each mouse in the experimental groups along with seven nonirradiated controls. Microarray analyses were performed as described in the Methods. We identified genes whose expression most highly correlated with exposure to radiation at a particular dose. Using a filtered gene list of 2,213 transcripts, we first performed an unsupervised analysis of the gene expression data from all samples to determine if there was structure evident in the expression information that reflected radiation exposure. Indeed, as shown in Figure 2A, an analysis of the data by hierarchical clustering revealed clear patterns of gene expression that separated the nonirradiated samples from the irradiated samples. Moreover, it was also evident that the clusters separated the samples as a function of radiation dose. These conditions of radiation exposure resulted in distinct gene expression events.

Given the evident patterns of gene expression reflecting exposure to radiation, we then used a supervised binary regression analysis to specifically focus on those patterns of gene expression, or what we term metagenes, that best classified and predicted the event of radiation exposure. Based upon a filtered gene list of 6,793 probes, a pattern of gene expression could be identified that effectively distinguished the control animals from those that were irradiated (Figure 2B). A critical aspect of these analyses is the ability to validate the classifications, testing that a pattern reflecting the radiation response does indeed have the capacity to actually predict the status of an unknown sample as opposed to being merely a chance association. To verify that these patterns did indeed represent genes reflecting the exposure to radiation, we utilized a leave-one-out cross-validation to assess the ability of the pattern to predict the relevant samples (Figure 2B, right). The results demonstrate that the pattern selected for distinguishing control animals from those irradiated at various doses does indeed have the capacity to predict the status of these samples. We conclude from these results that it is possible to identify a gene expression signature that reflects a response to radiation.

### Radiation Dose-Specific Gene Expression Signatures

**Figure 3:**
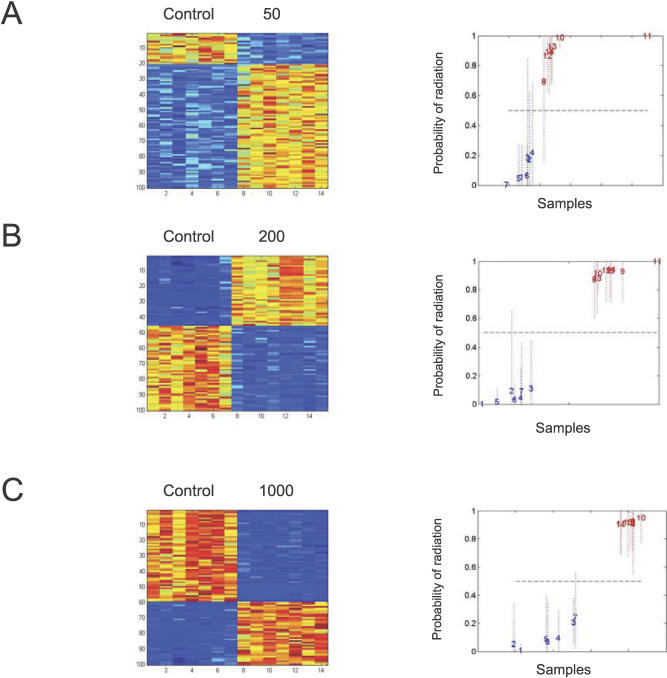
Gene Expression Profiles that Predict Dose of Radiation Exposure The left images depict the expression pattern of genes selected for classifying control, nonirradiated samples versus 50-cGy (A), 200-Gy (B), or 1,000-cGy (C) exposure. The right graphs depict a leave-one-out cross validation of the classification probabilities of control versus 50 cGy (A), control versus 200 cGy (B), or control versus 1,000 cGy (C). High expression is depicted as red, and low expression is depicted as blue; the relative expression levels for 0 versus 50 cGy range from 0.29 to 10.5, 0 versus 200 cGy range from 0.26 to 26.3, and 0 versus 1,000 cGy range from 0.17 to 48.77. Each sample is represented as a number in the chronological order of the dosed sample.

Given the ability to develop a signature reflecting radiation response, we proceeded to determine if expression signatures could be identified that were specific to the actual level of radiation. As shown in Figure 3A–3C (left), a series of profiles was identified that distinguished the control samples from samples irradiated at increasing doses. And again, each of these profiles was then validated via leave-one-out cross validation analysis, demonstrating the capacity to accurately predict the status of a sample treated as an unknown (Figure 3A–3C, right). This is most clear when distinguishing controls from 200 cGy or 1,000 cGy exposures (Figure 3B and 3C), possibly reflecting more pronounced effects on transcription at these higher dose exposures. Nevertheless, the results are quite clear that there are patterns that can be discerned with an ability to predict exposure to radiation.

The profiles that can distinguish between a control animal and one exposed to various doses of radiation are based on a collection of 75–100 genes per profile. A complete list of these discriminatory genes (annotated genes and ESTs) is provided in Table S1. The gene ontology categories represented by the genes selected for distinguishing various levels of radiation exposure are summarized in Table S2. The most significant annotations are shown in order of decreasing significance. Of note, while 21 annotated genes were overexpressed in common between the 200 cGy and 1,000 cGy exposure levels, only two annotated genes, *etoposide-induced 2.4 mRNA (Ei24), protein-phosphatase 1 regulatory inhibitor subunit 2 (Ppp1r2),* and three nonannotated genes, *M300006426, M300017997, M300015969,* were found to be in common across the three different profiles (Figure S1). Quantitative real-time PCR analysis confirmed the integrity of the gene expression levels obtained in the gene arrays (Figure S1). Moreover, a dose-response effect was not evident in the majority of genes within the metagene profiles; for example, of the 100 genes within the profile of 50-cGy exposure, only 12 (12%) demonstrated a further increase in expression at 200 cGy and 1,000 cGy. Taken together, these data suggest that each different level of radiation exposure induced a unique hierarchy of transcriptional events, rather than an escalating biological response from a select group of genes. The overexpression of *Ei24* in all three profiles suggests the integrity of these predictors, since this is a pro-apoptotic factor that would be expected to be up-regulated in response to ionizing radiation exposure [31]. Taken together, these results suggest that the response does reflect biological events associated with radiation and that distinct biological processes are activated as a function of radiation dose.

**Figure 4:**
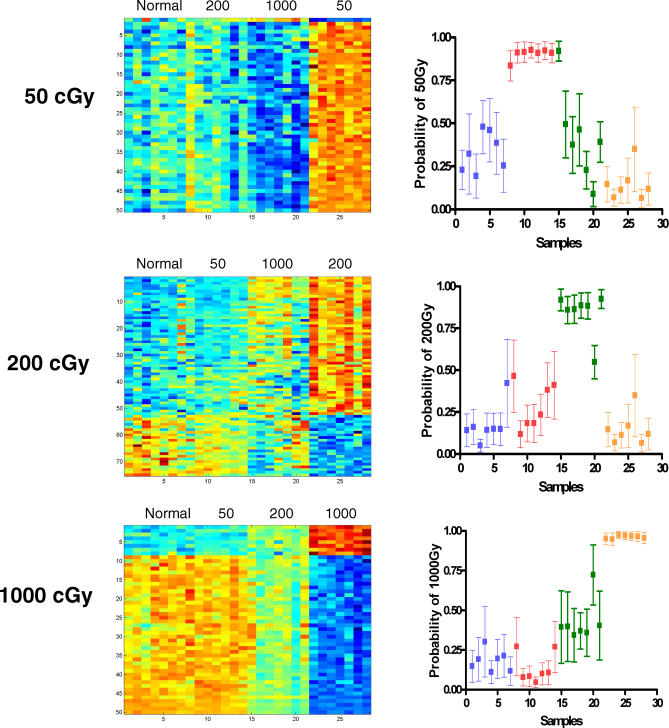
Gene Expression Profiles Can Distinguish Different Levels of Radiation Exposure To internally validate the metagene profiles, the predictive capacity of each profile was analyzed with regard to distinguishing nonirradiated samples from each of the irradiated samples. The heatmaps in the left depict the expression profiles of genes selected to discriminate the dose of radiation; high expression is depicted as red, and low expression is depicted as blue; the ranges of relative expression levels are the same as in Figure 3: 0 versus 50 cGy range from 0.29 to 10.5, 0 versus 200 cGy range from 0.26 to 26.3, and 0 versus 1,000 cGy range from 0.17 to 48.77. The right graphs depict a leave-one-out cross validation analysis to demonstrate that in each case, the profiles developed for a particular radiation dose predicted the relevant samples with a high level of accuracy. The samples from normal (nonirradiated) mice are represented as blue, samples from 50-cGy irradiated are red, samples from 200-cGy irradiated are green, and samples from 1,000-cGy irradiated mice are orange. As shown, the predictors for 50-cGy, 200-cGy, and 1,000-cGy irradiation misclassified only one sample out of 28 analyzed in each case, indicating a high level of accuracy of prediction.

The fact that the gene expression profiles selected for predicting each dose of radiation were largely nonoverlapping suggested that these were distinct profiles and thus suggested the potential for developing predictors that could not only distinguish radiation from control but that could also distinguish the dose of radiation. To address this possibility, we redeveloped the predictors of radiation response by focusing on the development of profiles that would distinguish a particular dose of radiation from not only control samples but also from each of the other irradiated samples. As shown in Figure 4 (left), expression profiles were identified that distinguished 50 cGy, 200 cGy, and 1,000 cGy samples not only from control samples but also from each of the other irradiated samples. A leave-one-out cross-validation analysis confirmed the effectiveness of these profiles toward distinguishing one level of radiation exposure versus the other levels (Figure 4, right). In each case, the profiles developed for a particular radiation dose proved to be highly accurate in predicting the relevant samples, with only one incorrectly categorized sample from *n* = 28 analyzed in each experiment.

### A Radiation Response Signature Can Predict Radiation Exposure in Human Samples

**Figure 5:**
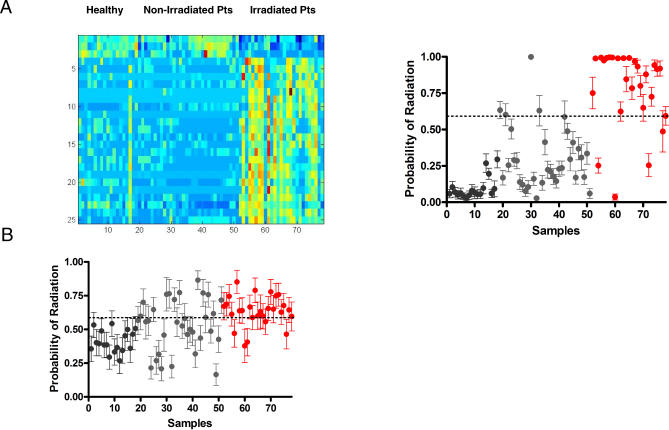
Metagene Profiles Generated in Mice and Humans Can Predict Radiation Exposure (A) A metagene profile of human radiation exposure generated in humans is represented. The heatmap on the left depicts the expression profiles of genes (rows) selected to discriminate the human samples (columns); high expression is depicted as red, and low expression is depicted as blue with the relative expression level ranging from 0.06–139.0. Healthy, healthy donors; Non-Irradiated Pts, nonirradiated patients; Irradiated Pts, irradiated patients. In the right image samples from irradiated patients are shown in red, whereas the samples from nonirradiated patients (gray) and healthy donors (black) are shown. The human metagene profile correctly distinguished 90% (85% sensitivity and 94% specificity) of the irradiated human samples versus the nonirradiated human samples. (B) A metagene profile of human radiation exposure generated in mice is represented. Samples from irradiated patients are shown in red, whereas the samples from nonirradiated patients (gray) and healthy donors (black) are shown. In an external validation study, the metagene profile of 1,000 cGy from the mouse successfully identified 77% (82% sensitivity and 75% specificity) of the irradiated human samples versus the nonirradiated human samples.

As a complementary approach, we sought to develop a metagene profile of human radiation exposure solely using PB samples collected from healthy donors and stem cell transplant patients prior to and six hours following TBI with 150 cGy or 200 cGy. For these studies, 22 patients were enrolled who received 200 cGy as a single fraction prior to a nonmyeloablative transplant. A total of nine patients were enrolled who received myeloablative fractionated irradiation (1,350 cGy) prior to allogeneic stem cell transplant, and five patients were enrolled who received myeloablative fractionated TBI (1,200 cGy) prior to autologous transplant. PB samples were also collected from three patients prior to receiving nonradiation-based conditioning as well as 18 healthy donors. The clinical characteristics of the enrolled patients are summarized in Table S3. Sufficient RNA was isolated from all 18 healthy human donors, 33 patients prior to receiving TBI or high dose chemotherapy conditioning, and 27 irradiated patients. For the gene expression analysis, a filtered human gene list of 11,319 probes was utilized. A supervised binary regression analysis identified a metagene profile of 25 genes (Figure 5A), which effectively distinguished the nonirradiated human samples from those from irradiated patients. A leave-one-out cross-validation analysis confirmed that this profile correctly predicted the human samples with an overall accuracy of 90%, a sensitivity of 85%, and a specificity of 94% (Figure 5A). Analysis of the 20 patients from whom both pre- and postirradiation samples were available demonstrated that the human metagene profile accurately predicted 18 of 20 (90%) preirradiation samples and 17 of 20 (85%) postirradiation samples. A Mann-Whitney log rank test was performed, which demonstrated a highly significant difference between the predicted probabilities of the healthy donors, nonirradiated patients, and the irradiated patients (*p* < 0.0001). The list of genes contained within the human predictor is provided in Table S4. The gene ontology categories represented by the human genes selected for distinguishing various levels of radiation exposure are summarized in Table S5.

To extend this analysis further, we asked if the predictive signature developed in the mouse could cross over to predict radiation exposure in humans. The potential advantage of this approach is the utility of the mouse in developing dose-specific signatures, something not easily achievable with human participants. For these studies, the same patient samples analyzed in Figure 5A were employed as a validation set for the mouse predictors. A total of 7,101 probes were mapped between the mouse and human operon probe sets and were used in validating the mouse model in the human samples. Of the three mouse predictors, the human homolog of 1,000 cGy (271 genes) showed the highest accuracy of prediction of the human samples. This predictor identified the human samples with an overall accuracy of 77%, sensitivity of 82%, and specificity of 75% (Figure 5B). Interestingly, the mouse predictor was 100% accurate (18/18) in identifying the samples from healthy donors as nonirradiated, but less accurate in distinguishing samples from pre-stem-cell transplant patients as nonirradiated (20/33, 61%). A Mann-Whitney log rank analysis demonstrated a highly significant difference between the predicted probabilities of the samples from healthy donors, nonirradiated patient samples, and the samples from irradiated patients (*p* < 0.0001). Taken together, these results indicate that this metagene profile of radiation response developed in the mouse performed exceptionally well in identifying healthy individuals as nonirradiated but less well in individuals with complex and advanced malignancies who had received extensive prior chemotherapy and radiotherapy. However, when this heterogeneous patient population underwent acute (therapeutic) radiation exposure, the accuracy of the metagene profile increased, demonstrating the sensitivity of the profile to detect 150- or 200-cGy exposure even within a markedly heterogeneous human population. Of note, only one gene, *protein kinase C-eta (PRKCH),* an anti-apoptotic factor [32], was found to be in common between the human derived predictor (25 genes) and the human homolog of the mouse 1,000 cGy predictor (271 genes). These data suggest that certain aspects of the molecular response to radiation may be very different in rodents compared to humans.

## Discussion

The ability to assess exposure to ionizing radiation, both to measure adverse effects that might result from low level exposure and to assess the extent of exposure following exposure to high-level radiation, could have a significant impact on subsequent clinical care. With this in mind, we have sought to develop gene expression signatures that could reflect radiation exposure and have the capacity to discriminate based on the exposure level. More broadly, these studies have the potential to identify specific genes and pathways involved in radiation-induced cellular damage and, therefore, targets for radioprotective intervention. An examination of the functional categories represented within the mouse radiation profiles revealed protein and cellular biosynthesis and immune response as highly represented gene predictor functions (Table S2). Additionally, individual genes involved in DNA repair (e.g., *recombination activating gene 1 [Rag1]*) [33] as well as hematopoietic cell activation *(membrane spanning 4-domains, subfamily A, member 1 [Ms4a1]*) [34] were identified. More generally, biological processes including apoptosis (e.g., *Ei24*) were highlighted. While it may be premature to consider how the biological processes represented by the radiation response genes we have identified may be exploited for therapeutic purposes, these results clearly provide clues to guide future studies focused on this goal.

A more practical application of these methods can be seen in the context of heightened concerns regarding the risk of terrorist-mediated attacks using radiological or nuclear weapons. These articulated concerns have prompted renewed focus on the development of countermeasures to the effects of ionizing radiation injury [10–13]. Experimental models have demonstrated that many interventions aimed at ameliorating the effects of radiation depend on the early administration of such therapies (e.g., cytokine administration for marrow protection), after which their effectiveness wanes [20,35,36]. In the event of a “dirty bomb” or detonation of an “improvised nuclear device,” it is clear that thousands to tens of thousands of individuals, many of whom will represent the “worried well,” will present for medical evaluation [10–13] as occurred following the accidental Cs137 exposure in Goiania, Brazil [37]. Since the symptoms of ionizing radiation exposure can be mild to absent within the first days to weeks, it would be difficult for health care professionals to distinguish those truly exposed from the “worried well” without a rapid and sensitive test to make this distinction. In this study, we show that patterns of gene expression changes (metagenes) in the PB can be identified that distinguish medically relevant levels of radiation exposure. For example, the metagene profile we developed for 0-cGy exposure (nonexposed) demonstrated 100% accuracy in distinguishing nonexposed mice from those exposed to as little as 50 cGy. This approach therefore has the potential to facilitate the correct identification of the “worried well” from those even with relatively low-level radiation exposure, which would be a critical first step in any mass casualty event. Not surprisingly, the metagene profiles we developed to predict higher levels of radiation exposure (200 cGy and 1,000 cGy) provided more obvious distinction than the 50-cGy profile with regard to distinguishing any level of irradiation versus nonirradiated samples.

The approach we have taken to utilize gene expression profiles to predict radiation exposure has been further validated by our human studies in which a metagene profile generated solely from human samples demonstrated an accuracy of 90% in distinguishing healthy donors, nonirradiated patients, and irradiated patients. Given the heterogeneous nature of the human population we have studied, this level of accuracy of the human predictor in distinguishing irradiated and nonirradiated patients was encouraging. The fact that a highly accurate predictor could be generated from the human samples also demonstrates the potential for identifying relevant information in human PB samples. We plan to externally validate this predictor against a larger cohort of irradiated patients and anticipate that further analysis and refinement of these human signatures could generate a predictor of radiation response with substantial accuracy. Comparison of this human metagene profile of radiation response with a set of human radiation-induced biomarkers described by Amundson et al. [18] revealed only one transcript, DNA-damage binding-protein 2, which was overexpressed in both studies. The lack of overlap between these two studies may reflect differences in gene representation on the different arrays utilized (Human Operon array representing 24,650 genes versus a 6,485-element cDNA array) or the different number of patient samples that underwent array in each study (60 patients versus one patient) [18].

As further proof-of-principle of the potential application of these predictive tools to human patients, we sought to prospectively test the metagene profiles of radiation response developed in mice against PB samples collected from both healthy volunteers and stem cell transplantation patients prior to and six hours following TBI with 150 cGy or 200 cGy. The mouse predictor of 1,000 cGy demonstrated an overall accuracy of 77% in predicting the human PB samples correctly. Given that this is the first study, to our knowledge, to attempt to apply a metagene profile to predict radiation response in humans and the heterogeneous nature of the patients being studied, we believe this level of accuracy is encouraging. Moreover, the 100% accuracy of the mouse predictor in identifying 18 healthy donors as “nonirradiated” suggests the potential utility of this approach in screening healthy members of a given community for radiation exposure. Conversely, the lower level of accuracy of the mouse predictor in identifying nonirradiated transplant patients as “nonirradiated” (61%) indicates that further refinement of this signature will be necessary and that such an approach may have difficulty when testing individuals with complex diseases or those undergoing medical therapies. Since a variety of hematological diseases were represented in this patient cohort, and 37 of the 39 patients (95%) had received some form of cytotoxic chemotherapy prior to enrollment, we were unable, in this study, to measure the impact of these two variables on gene expression in these patients. We anticipate that enrollment of additional patients over time will allow the impact of these factors to be more formally measured.

The relative radioresistance of rodents compared to humans also may have impacted the accuracy of the mouse-metagene profiles when applied against human samples [38–41]. For example, several studies have demonstrated that primary human cells in culture are 2- to 4-fold more sensitive to the deleterious effects of ionizing radiation as compared to rodent cells in culture [38–41]. In this study, the 200-cGy mouse-metagene profile successfully distinguished only 62% of the 200-cGy irradiated human samples, whereas the mouse 1,000-cGy metagene profile demonstrated substantially better accuracy (77%). Examination of additional doses of radiation in mice over time will provide the potential for further refinement of the mouse predictor as applied against human samples. Moreover, since the mouse-metagene profiles described here were generated from female, adult mice from a single strain, we recognize that additional studies of both genders, young and old mice, multiple strains, and the impact of time on the stability of the metagene profiles will be important for further refinement of this approach. Nonetheless, the fact that a mouse profile could predict human radiation exposure suggests that further refinement of the mouse signature, to include profiles derived from higher doses, could be effective in generating a dose-specific predictor of human radiation exposure.

Finally, we return to what we believe represents a more general implication of this work. Although prediction of radiation exposure can be viewed as a practical goal, radiation exposure can also be seen more generally as an “environmental exposure,” which now can be quantified. In principle, it should be possible to extend the concept more broadly to a variety of other relevant environmental agents, such as toxins, carcinogens, and others that have a significant impact on human phenotypes. An ability to provide a quantitative measure of the environmental contribution to various phenotypes, along with measures of the genetic contribution, could enhance the ability to more accurately describe and understand these phenotypes.

## Supporting Information

Figure S1 Distinct Gene Expression Profiles Are Activated as a Function of Radiation Dose (A) A Venn diagram of three mouse-metagene profiles is shown. The diagram illustrates the number of genes within the 50-cGy, 200-cGy, and 1,000-cGy mouse-metagene profiles and the intersection of genes represented in common between the three profiles. Only five genes were found to be in common between the three metagene profiles, suggesting that distinct biological processes were activated as a function of radiation dose.
(B) Quantitative real-time PCR analysis of representative genes within the metagene profiles is shown. The fold changes in expression of *Ei24* (top) and *Ptprcap* (bottom) in the PB of irradiated mice are shown relative to the nonirradiated control group. Error bars indicate standard deviation. The levels of expression of these genes correlated with that observed in the gene array analysis. A dose-response to increasing radiation dose was observed with *Ei24* but not with *Ptprcap*.
(41 KB PPT)

Table S1 Genes that Distinguish Radiation Responses in Mice (342 KB DOC)

Table S2 Top Gene Predictor Functions Found within the Mouse Prediction Model Genes that were defined in the metagenes were annotated in GATHER (http://meddb01.duhs.duke.edu/gather/), which quantifies the evidence supporting the association between a gene group and an annotation using a Bayes factor [41]. This assesses the hypothesis that the distribution of annotations varies across gene groups against the hypothesis that the distribution is identical. A positive Bayes factor indicates that the evidence supports the association, while a negative one indicates no association. Its magnitude corresponds to the strength of the evidence for the association, where higher values are stronger (ln[Bayes factor]). The ten most significant ontology annotations are shown in order of decreasing significance. The most highly represented biological processes for each radiation dose are shown in the left column. The middle column shows the number of genes within the metagene against the number of annotated genes within the mouse genome. The far right column shows the Bayes factor and those genes within the radiation signature that are found within the biological process.
(55 KB DOC)

Table S3 Patient Characteristics (26 KB DOC)

Table S4 Genes that Distinguish Radiation Response in Humans (45 KB DOC)

Table S5 Top Gene Predictor Functions Found within the Human Prediction Model Genes that were defined in the human radiation metagenes were annotated in GATHER (http://meddb01.duhs.duke.edu/gather/) [41] and represented as described in Table S2.
(45 KB DOC)

### Accession Numbers

The Gene Expression Omnibus (GEO [http://www.ncbi.nlm.nih.gov/geo]) accession number for the microarray data from this study is GSE6874.

## Abbreviations

MNC: mononuclear cell
PB: peripheral blood
TBI: total body irradiation
