# Supplementary material for: Gene Expression Signatures That Predict Radiation Exposure in Mice and Humans
Source: PLoS Med. 2007 Apr 3;4(4):e106. doi: 10.1371/journal.pmed.0040106 (PMC1845155; doi:10.1371/journal.pmed.0040106)
Supplement: Supplementary file 1 [file pmed.0040106.st001.doc]

**Supplemental Table 1. Genes that distinguish radiation responses.**

**Operon oligo ID can be queried in the OMAD database (http://omad.operon.com/mouseV3/query.php)**

| **Operon Oligo ID** | **Gene Symbol** | **RefSeq** | **Genbank** |
| --- | --- | --- | --- |
| **50 Gy** |  |  |  |
| M200000096 | B3Gat3 | NM_024256 | BC002103 |
| M200000793 | Syngr2 | NM_009304 | AF151985 |
| M200001123 | AF006998 | -- | AK010234 |
| M200001264 | Ramp1 | -- | AJ314840 |
| M200002378 | S100a13 | NM_009113 | BC005687 |
| M200003512 | Cgbp-pending | NM_028868 | AK010337 |
| M200003784 | Bax | NM_007527 | L22472 |
| M200003927 | Pum2 | NM_030723 | AY027917 |
| M200004382 | B230365D05Rik | NM_146124 | BC006592 |
| M200004816 | Bin3 | NM_021328 | AK040685 |
| M200004967 | S100a1 | NM_011309 | AK002721 |
| M200005113 | 2310003C10Rik | NM_024209 | BC002223 |
| M200005348 | hypothetical protein | NM_145410 | BC008155 |
| M200005569 | Arh-pending | NM_145554 | BC021467 |
| M200006558 | Bcdin3 | NM_144913 | BC017157 |
| M200006610 | D4Wsu114e | NM_133873 | BC017525 |
| M200006734 | 1500002O20Rik | NM_028047 | BC034211 |
| M200006844 | Man2c1 | -- | BC016253 |
| M200008056 | Rfxap | NM_133231 | AF335512 |
| M200011599 | Mycbpap | NM_153807 | AK034209 |
| M200011886 | Nd1-pending | NM_054102 | AB055737 |
| M200011988 | 0610007L01Rik | -- | AK002297 |
| M200014062 | D5Buc26e | -- | AK077934 |
| M200014198 | 1810059G22Rik | NM_026325 | AK010996 |
| M200014359 | 2610307O08Rik | -- | AK012006 |
| M200015951 | Olfr1509 | NM_020514 | AY073295 |
| M300000109 | Rpl13 | NM_016738 | AK010989 |
| M300000424 | Rps11 | NM_013725 | AK011207 |
| M300000487 | Bax | NM_007527 | L22472 |
| M300000561 | D6Ertd772e | NM_025816 | BC014798 |
| M300000753 | Snf8 | NM_033568 | BC003938 |
| M300001996 | Sara | NM_009120 | L20294 |
| M300002291 | -- | -- | -- |
| M300002851 | -- | -- | -- |
| M300003559 | Btg3 | NM_009770 | D83745 |
| M300003570 | Eif4a2 | NM_013506 | U64706 |
| M300003995 | Rps10 | NM_025963 | X13549 |
| M300005874 | C330027G06Rik | -- | AK015041 |
| M300006426 | -- | -- | -- |
| M300007102 | Kras2 | NM_021284 | X00485 |
| M300007488 | Taf10 | NM_020024 | AJ249987 |
| M300007924 | Taf1c | NM_021441 | Y09974 |
| M300008054 | Pts | NM_011220 | BC029013 |
| M300008077 | Ei24 | NM_007915 | U41751 |
| M300008187 | 2300004C15Rik | NM_027086 | AK009025 |
| M300008242 | 2010004J23Rik | -- | BC003459 |
| M300008257 | AI481279 | NM_133978 | AF188504 |
| M300009966 | Sui1-rs1 | NM_011508 | Z50159 |
| M300010063 | -- | -- | -- |
| M300010228 | Galt | NM_016658 | U41282 |
| M300010329 | 1110014N07Rik | NM_023162 | AF230339 |
| M300010693 | 2010203J19Rik | NM_025919 | BC021402 |
| M300010729 | -- | NM_153140 | BC023364 |
| M300010806 | Mospd3 | NM_030037 | -- |
| M300012862 | -- | -- | -- |
| M300012951 | Rps19 | -- | AK012052 |
| M300013807 | -- | -- | -- |
| M300013912 | Rpl9 | NM_011292 | U17331 |
| M300013994 | -- | -- | -- |
| M300014119 | -- | -- | -- |
| M300014454 | -- | -- | -- |
| M300014487 | Ppp1r2 | NM_025800 | AK053657 |
| M300014509 | -- | -- | -- |
| M300014689 | Rps19 | NM_023133 | -- |
| M300015084 | -- | -- | -- |
| M300015153 | -- | -- | -- |
| M300015404 | Rps3a | NM_016959 | AK003158 |
| M300015601 | -- | -- | -- |
| M300015969 | -- | -- | -- |
| M300016018 | -- | NM_023133 | -- |
| M300016278 | -- | -- | -- |
| M300016629 | -- | -- | -- |
| M300016640 | -- | -- | -- |
| M300016945 | H3f3b | NM_008211 | BC021768 |
| M300016997 | -- | -- | -- |
| M300017556 | -- | -- | -- |
| M300017934 | -- | -- | -- |
| M300017997 | -- | -- | -- |
| M300018759 | -- | -- | -- |
| M300018897 | -- | -- | -- |
| M300018940 | -- | -- | -- |
| M300018952 | -- | -- | -- |
| M300019090 | Rpl29 | NM_009082 | L08651 |
| M300019110 | -- | -- | -- |
| M300019243 | -- | -- | -- |
| M300019345 | -- | -- | -- |
| M300019355 | -- | -- | -- |
| M300019356 | -- | -- | -- |
| M300019634 | -- | -- | -- |
| M300019659 | -- | -- | -- |
| M300019689 | -- | -- | -- |
| M300020488 | -- | -- | V00754 |
| M300020556 | -- | -- | -- |
| M300020566 | -- | -- | -- |
| M300020718 | -- | -- | -- |
| M300020857 | Rps16 | NM_013647 | M11408 |
| M300021242 | -- | -- | -- |
| M300021555 | -- | -- | -- |
| M300021619 | -- | -- | -- |
| M300021638 | -- | -- | -- |
|  |  |  |  |
| **200 Gy** |  |  |  |
| M200005905 | -- | -- | BC022623 |
| M200007177 | -- | -- | -- |
| M200008262 | -- | -- | BC018495 |
| M200009525 | -- | -- | -- |
| M200013719 | 2010002N04Rik | NM_134133 | BC012898 |
| M300006426 | -- | -- | -- |
| M300006953 | -- | -- | -- |
| M300007066 | -- | -- | -- |
| M300008551 | -- | -- | -- |
| M300010073 | -- | -- | -- |
| M300011495 | -- | -- | BG088667 |
| M300012450 | -- | -- | -- |
| M300014084 | -- | -- | -- |
| M300015511 | -- | -- | -- |
| M300015969 | -- | -- | -- |
| M300016183 | -- | -- | BC022753 |
| M300016406 | -- | -- | -- |
| M300016870 | -- | -- | -- |
| M300017080 | -- | -- | -- |
| M300017864 | -- | -- | -- |
| M300017997 | -- | -- | -- |
| M300018207 | -- | -- | -- |
| M300018341 | -- | -- | -- |
| M300018546 | -- | -- | -- |
| M300018869 | -- | -- | -- |
| M300019345 | -- | -- | -- |
| M300019589 | -- | -- | -- |
| M300019860 | -- | -- | -- |
| M300020088 | -- | -- | -- |
| M300020351 | -- | -- | -- |
| M300020771 | -- | -- | -- |
| M300021174 | -- | -- | -- |
| M300021430 | -- | -- | -- |
| M300022080 | -- | -- | -- |
| M200006174 | 0610039P13Rik | NM_028752 | BC021548 |
| M200006698 | 1110003B01Rik | NM_026131 | AK003338 |
| M300011249 | 1110007C09Rik | NM_026738 | AK003526 |
| M200006445 | 2010110M21Rik | NM_025933 | BC021471 |
| M300002788 | 2200005K02Rik | NM_145975 | BC026492 |
| M300002728 | 2210008F15Rik | NM_023554 | AB041566 |
| M300017554 | 4930415K17Rik | NM_133687 | BC016207 |
| M300002970 | 5730420B22Rik | NM_172597 | AK017582 |
| M300015464 | Actc1 | NM_009608 | M26774 |
| M300014099 | Actl | NM_013798 | AF195094 |
| M200001333 | Adss1 | NM_007421 | M74495 |
| M200003779 | Alox5ap | -- | BC026209 |
| M200004869 | BC019776 | NM_144797 | AK034855 |
| M300009582 | BC031353 | NM_153584 | BC031353 |
| M300006168 | Cc2d1b | NM_177045 | -- |
| M200015923 | Cd72 | NM_007654 | S40777 |
| M200001144 | Cd79b | NM_008339 | AF002279 |
| M300003410 | Chkl | NM_007692 | AB011000 |
| M300012025 | Ciz1 | NM_028412 | BC018483 |
| M200000788 | Ctps2 | NM_018737 | AK084595 |
| M300008077 | Ei24 | NM_007915 | U41751 |
| M300003570 | Eif4a2 | NM_013506 | U64706 |
| M200004470 | Fcer1g | NM_010185 | J05020 |
| M200009330 | Fgls-pending | NM_025378 | AY082484 |
| M300004256 | Fth | NM_010239 | M24509 |
| M200006815 | Gabarap | NM_019749 | BC030350 |
| M300006113 | Glipr2 | -- | BC031750 |
| M300010992 | H2-DMb1 | NM_010388 | X62743 |
| M300010374 | Hpcal1 | NM_016677 | D13126 |
| M300010905 | Icam1 | NM_010493 | M90550 |
| M300014777 | Ifitm3l | NM_030694 | AY082486 |
| M300005305 | Lcn2 | -- | X81627 |
| M200003020 | Limd1 | NM_013860 | AJ132409 |
| M200004061 | Litaf | NM_019980 | AK076162 |
| M200006408 | Lmo2 | NM_008505 | M64360 |
| M200000312 | Ly6d | NM_010742 | L40419 |
| M200005409 | Metapl1 | NM_025633 | AK005241 |
| M200009488 | Midn | NM_021565 | BC034719 |
| M200000897 | Mll | -- | L17069 |
| M300004265 | Ms4a1 | NM_007641 | AK017903 |
| M200011886 | Nd1-pending | NM_054102 | AB055737 |
| M200002750 | Nfkbia | NM_010907 | U57524 |
| M200002585 | Oas2 | NM_145227 | AF418010 |
| M300014487 | Ppp1r2 | NM_025800 | AK053657 |
| M300004391 | Prdx5 | NM_012021 | BC008174 |
| M200013567 | Ptprcap | NM_016933 | BC013273 |
| M300007368 | Rab6 | NM_024287 | M79313 |
| M200001765 | Rbbp6 | NM_011247 | U28789 |
| M300005864 | Rga | NM_009057 | X96618 |
| M300000976 | Rps18 | NM_011296 | AB000911 |
| M300016018 | Rps19 | NM_023133 | -- |
| M300015404 | Rps3a | NM_016959 | AK003158 |
| M200003339 | Sdcbp | NM_016807 | BC019400 |
| M300015731 | Serpinb1a | NM_025429 | AK008914 |
| M300003571 | Siat1 | NM_145933 | D16106 |
| M300007254 | Siglec10 | NM_172900 | -- |
| M300010226 | Slc2a6 | NM_172659 | -- |
| M300004665 | Taldo1 | -- | U63159 |
| M200013739 | Tfam | NM_009360 | L07107 |
| M300013351 | Tpp2 | NM_009418 | X81323 |
| M300021404 | Vamp8 | NM_016794 | BC012668 |
| M200004735 | Wbscr5 | NM_022964 | AF139987 |
| M300005643 | Xrn2 | NM_011917 | AK053643 |
| M300006355 | Yrdc | NM_153566 | -- |
| M200003641 | Zfp36l1 | NM_007564 | M58566 |
| M200002019 | Zyx | NM_011777 | X99063 |
|  |  |  |  |
| **1000 Gy** |  |  |  |
| M200008262 | -- | -- | BC018495 |
| M200009525 | -- | -- | -- |
| M200013719 | 2010002N04Rik | NM_134133 | BC012898 |
| M200014574 | -- | -- | -- |
| M300000976 | Rps18 | NM_011296 | AB000911 |
| M300002583 | -- | -- | -- |
| M300006168 | Cc2d1b | NM_177045 | -- |
| M300006355 | Yrdc | NM_153566 | -- |
| M300006426 | -- | -- | -- |
| M300006455 | Pank4 | NM_172990 | -- |
| M300006953 | -- | -- | -- |
| M300007254 | Siglec10 | NM_172900 | -- |
| M300008551 | -- | -- | -- |
| M300009622 | 0610008L10Rik | NM_144830 | BC022145 |
| M300010073 | -- | -- | -- |
| M300010374 | Hpcal1 | NM_016677 | D13126 |
| M300011495 | -- | -- | BG088667 |
| M300011848 | E430018M08Rik | NM_173445 | -- |
| M300012334 | Actg1 | NM_009609 | M26111 |
| M300013208 | Eif2c1 | NM_153403 | -- |
| M300014084 | -- | -- | -- |
| M300014256 | -- | -- | -- |
| M300014569 | -- | -- | -- |
| M300014614 | -- | -- | -- |
| M300014853 | -- | -- | -- |
| M300015742 | -- | -- | M37736 |
| M300015969 | -- | -- | -- |
| M300016042 | -- | -- | -- |
| M300016183 | -- | -- | BC022753 |
| M300016406 | -- | -- | -- |
| M300016870 | -- | -- | -- |
| M300017080 | -- | -- | -- |
| M300017600 | -- | NM_146237 | BC024537 |
| M300017732 | -- | -- | -- |
| M300017740 | -- | -- | -- |
| M300017997 | -- | -- | -- |
| M300018207 | -- | -- | -- |
| M300018526 | -- | -- | -- |
| M300018546 | -- | -- | -- |
| M300018549 | -- | -- | -- |
| M300018869 | -- | -- | -- |
| M300019445 | -- | -- | -- |
| M300019589 | -- | -- | -- |
| M300019860 | -- | -- | -- |
| M300020049 | -- | -- | -- |
| M300020088 | -- | -- | -- |
| M300020351 | -- | -- | -- |
| M300020459 | -- | -- | -- |
| M300020491 | -- | -- | U38498 |
| M300021619 | -- | -- | -- |
| M300021787 | -- | -- | -- |
| M300022080 | -- | -- | -- |
| M200006174 | 0610039P13Rik | NM_028752 | BC021548 |
| M200006698 | 1110003B01Rik | NM_026131 | AK003338 |
| M300011249 | 1110007C09Rik | NM_026738 | AK003526 |
| M200008116 | 1200015A22Rik | NM_028766 | BC024933 |
| M200006445 | 2010110M21Rik | NM_025933 | BC021471 |
| M300010982 | 2310035K24Rik | NM_027129 | AK009634 |
| M200015240 | 2310044D20Rik | NM_026321 | AK020633 |
| M200006325 | 2410004M09Rik | NM_028035 | BC010334 |
| M300017554 | 4930415K17Rik | NM_133687 | BC016207 |
| M200006227 | 5830446M03Rik | NM_133934 | AK017996 |
| M200004076 | A930014I12Rik | NM_144542 | BC008220 |
| M300015464 | Actc1 | NM_009608 | M26774 |
| M200011659 | AI467246 | NM_133962 | BC009156 |
| M200003779 | Alox5ap | -- | BC026209 |
| M200001228 | Arhgef1 | NM_008488 | BC012488 |
| M200003784 | Bax | NM_007527 | L22472 |
| M300000487 | Bax | NM_007527 | L22472 |
| M300006464 | Ccnl2 | -- | AB041605 |
| M200015923 | Cd72 | NM_007654 | S40777 |
| M200001144 | Cd79b | NM_008339 | AF002279 |
| M200007578 | Cdkn1a | NM_007669 | U24173 |
| M300000077 | D15Ertd417e | NM_144811 | BC021398 |
| M200006364 | Dcxr | NM_026428 | AK004023 |
| M200003798 | Dgka | NM_016811 | BC006713 |
| M300008077 | Ei24 | NM_007915 | U41751 |
| M200004470 | Fcer1g | NM_010185 | J05020 |
| M300002629 | Gdi2 | NM_008112 | AK007871 |
| M300006113 | Glipr2 | -- | BC031750 |
| M200013566 | Gng12 | NM_025278 | AK005561 |
| M300010457 | H2-Aa | NM_010378 | X52643 |
| M300020898 | Lsm10-pending | NM_138721 | BC026476 |
| M200000312 | Ly6d | NM_010742 | L40419 |
| M200000279 | Ly6e | NM_008529 | U47737 |
| M200007568 | Madh7 | NM_008543 | AJ000551 |
| M200006275 | Mbc2 | -- | AF098634 |
| M200005409 | Metapl1 | NM_025633 | AK005241 |
| M300004265 | Ms4a1 | NM_007641 | AK017903 |
| M200003621 | Ninj1 | NM_013610 | AF219626 |
| M300014487 | Ppp1r2 | NM_025800 | AK053657 |
| M200013567 | Ptprcap | NM_016933 | BC013273 |
| M300005864 | Rga | NM_009057 | X96618 |
| M300015327 | Rps8 | NM_009098 | AK012562 |
| M200003339 | Sdcbp | NM_016807 | BC019400 |
| M300015731 | Serpinb1a | NM_025429 | AK008914 |
| M300003571 | Siat1 | NM_145933 | D16106 |
| M300021404 | Vamp8 | NM_016794 | BC012668 |
| M200001806 | Vil2 | NM_009510 | X60671 |
| M200004735 | Wbscr5 | NM_022964 | AF139987 |
