# Supplementary material for: Gene Expression Signatures That Predict Radiation Exposure in Mice and Humans
Source: PLoS Med. 2007 Apr 3;4(4):e106. doi: 10.1371/journal.pmed.0040106 (PMC1845155; doi:10.1371/journal.pmed.0040106)
Supplement: Supplementary file 2 [file pmed.0040106.st002.doc]

| **GO Ontology of predictor genes found in the  mouse radiation signature** | **Genes with GO term in list** | **Genes within  Mouse Genome with annotation in GO term** | **ln(Bayes factor)** | **Genes** |
| --- | --- | --- | --- | --- |
| **50 Gy** |  |  |  |  |
| protein biosynthesis | 11 | 465 | 14.79 | Eif4a2 Pum2 Rpl11 Rpl13 Rpl29 Rpl9 Rps11 Rps16 Rps19 Rps3a Sui1-rs1 |
| macromolecule biosynthesis | 11 | 518 | 13.72 | Eif4a2 Pum2 Rpl11 Rpl13 Rpl29 Rpl9 Rps11 Rps16 Rps19 Rps3a Sui1-rs1 |
| ribosome biogenesis | 6 | 80 | 13.07 | Rpl13 Rpl29 Rpl9 Rps16 Rps19 Rps3a |
| ribosome biogenesis and  assembly | 6 | 86 | 12.66 | Rpl13 Rpl29 Rpl9 Rps16 Rps19 Rps3a |
| cytoplasm organization  and biogenesis | 6 | 96 | 12.05 | Rpl13 Rpl29 Rpl9 Rps16 Rps19 Rps3a |
| cellular biosynthesis | 12 | 798 | 11.62 | Eif4a2 Pts Pum2 Rpl11 Rpl13 Rpl29 Rpl9 Rps11 Rps16 Rps19 Rps3a Sui1-rs1 |
| biosynthesis | 12 | 834 | 11.16 | Eif4a2 Pts Pum2 Rpl11 Rpl13 Rpl29 Rpl9 Rps11 Rps16 Rps19 Rps3a Sui1-rs1 |
| cellular macromolecule  metabolism | 17 | 2362 | 7.89 | 2300004C15Rik Bax Eif4a2 Galt H3f3b Ppp1r2 Ppp6c Pum2 Rpl11 Rpl13 Rpl29  Rpl9 Rps11 Rps16 Rps19 Rps3a Sui1-rs1 |
| macromolecule metabolism | 17 | 2479 | 7.25 | 2300004C15Rik Bax Eif4a2 Galt H3f3b Ppp1r2 Ppp6c Pum2 Rpl11 Rpl13 Rpl29  Rpl9 Rps11 Rps16 Rps19 Rps3a Sui1-rs1 |
| primary metabolism | 24 | 5032 | 6.42 | 2300004C15Rik Bax Cxxc1 D11Moh34 Eif4a2 Galt H3f3b Ppp1r2 Ppp6c Pts  Pum2 Rpl11 Rpl13 Rpl29 Rpl9 Rps10 Rps11 Rps16 Rps19 Rps3a Sui1-rs1 Taf10 Taf1c Znrd1 |
| **200 Gy** |  |  |  |  |
| antigen presentation, exogenous  antigen via MHC class II | 2 | 10 | 3.9 | Fcer1g H2-DMb2 |
| cellular biosynthesis | 8 | 802 | 2.81 | Adssl1 Ctps2 Eif4a2 Fcer1g Rps18 Rps19  Rps3a St6gal1 |
| antigen presentation, exogenous  antigen | 2 | 20 | 2.67 | Fcer1g H2-DMb2 |
| ribosome biogenesis | 3 | 83 | 2.64 | Rps18 Rps19 Rps3a |
| protein biosynthesis | 6 | 470 | 2.59 | Eif4a2 Fcer1g Rps18 Rps19 Rps3a St6gal1 |
| biosynthesis | 8 | 838 | 2.56 | Adssl1 Ctps2 Eif4a2 Fcer1g Rps18 Rps19  Rps3a St6gal1 |
| ribosome biogenesis  and assembly | 3 | 89 | 2.45 | Rps18 Rps19 Rps3a |
| interleukin-10 biosynthesis | 1 | 1 | 2.2 | Fcer1g |
| regulation of  interleukin-10 biosynthesis | 1 | 1 | 2.2 | Fcer1g |
| positive regulation of  interleukin-10 biosynthesis | 1 | 1 | 2.2 | Fcer1g |
| **1000 Gy** |  |  |  |  |
| regulation of organismal  physiological process | 4 | 108 | 5.07 | Actc1 Cdkn1a Fcer1g H2-Aa |
| positive regulation of immune response | 3 | 51 | 4.42 | Cdkn1a Fcer1g H2-Aa |
| positive regulation of organismal  physiological process | 3 | 54 | 4.26 | Cdkn1a Fcer1g H2-Aa |
| antigen presentation, exogenous  antigen via MHC class II | 2 | 10 | 4.24 | Fcer1g H2-Aa |
| organelle organization and biogenesis | 8 | 739 | 4.2 | Actc1 Actg1 Arhgef18 Bax Cbx7 Rps18 Rps8 Vil2 |
| positive regulation of cellular  physiological process | 4 | 174 | 3.35 | Bax Cdkn1a Fcer1g H2-Aa |
| regulation of immune response | 3 | 76 | 3.32 | Cdkn1a Fcer1g H2-Aa |
| cell organization and biogenesis | 8 | 875 | 3.15 | Actc1 Actg1 Arhgef18 Bax Cbx7 Rps18 Rps8 Vil2 |
| antigen presentation, exogenous  antigen | 2 | 20 | 3.01 | Fcer1g H2-Aa |
| small GTPase mediated signal  transduction | 4 | 192 | 2.99 | Arhgef1 Arhgef18 Gdi3 Sdcbp |
