# Supplementary material for: Gene Expression Signatures That Predict Radiation Exposure in Mice and Humans
Source: PLoS Med. 2007 Apr 3;4(4):e106. doi: 10.1371/journal.pmed.0040106 (PMC1845155; doi:10.1371/journal.pmed.0040106)
Supplement: Supplementary file 3 [file pmed.0040106.st003.doc]

Supplemental Table 3. Patient Characteristics

_______________________________________________________

Characteristic	Number

*Samples analyzed*	n=18 healthy, 33 patients (pre-RT), 27 patients (post-RT)

*Patient Age*	46.5 years (mean)

*Diagnoses*	MDS/AML	(n=10)

ALL (n=5)

Multiple myeloma (n=6)

CML (n=3)

Non-Hodgkin’s Lymphoma (n=9)

Hodgkin’s Disease (n=2)

Myelofibrosis (n=2)

Scleroderma (n=1)

Sickle cell disease (1)

*Total No. of Patients*	n=39

*Prior radiotherapy*	n=10

*Prior chemotherapy*	n=37

*Transplantation type*	Non-myeloablative allogeneic/200 cGy (n=22)

Myeloablative allogeneic/1350 cGy (n=9)

Myeloablative autologous/1200 cGy (n=5)

Myeloablative allogeneic/chemotherapy (n=3)

Patients undergoing either non-myeloablative or myeloablative allogeneic or autologous stem cell transplantation were eligible for enrollment. All irradiation conditioning was completed 18 hours prior to the initiation of additional immunosuppressive conditioning drugs. PB samples were collected prior to and 6 hours following 200 cGy total body irradiation (non-myeloablative conditioning) or prior to and 6 hours following the first fraction (150 cGy) of total body irradiation (myeloablative conditioning). Six samples collected from pre-irradiated patients and 12 samples collected from post-irradiated patients were found to have RNA of insufficient quality for gene hybridization.

Pre-RT=pre-radiotherapy, Post-RT=post-radiotherapy, MDS=myelodysplastic syndrome, AML=acute myelogenous leukemia, ALL=acute lymphocytic leukemia, CML=chronic myelogenous leukemia
