# Supplementary material for: Gene Expression Signatures That Predict Radiation Exposure in Mice and Humans
Source: PLoS Med. 2007 Apr 3;4(4):e106. doi: 10.1371/journal.pmed.0040106 (PMC1845155; doi:10.1371/journal.pmed.0040106)
Supplement: Supplementary file 4 [file pmed.0040106.st004.doc]

| **Human Operon ID** | **GeneSymbol** | **Description** | **RefSeq** | **GB_accession** |
| --- | --- | --- | --- | --- |
| H300019371 | DDB2 | damage-specific DNA binding protein 2, 48kDa | NM_000107 | U18300,BC050455 |
| H300016408 | DDB2 | damage-specific DNA binding protein 2, 48kDa | NM_000107 | BC050455,U18300 |
| H300021118 | BBC3 | BCL2 binding component 3 | NM_014417 | U82987,AF332558 |
| H300007276 | - | ribosomal protein S27-like | NM_015920 | - |
| H300007443 | - | ribosomal protein S27-like | NM_015920 | - |
| H300004296 | EST | - | - | BC065505 |
| H300008065 | EST | - | - | BC065505 |
| H300010830 | NP_073604 | hypothetical protein FLJ12484 | NM_022767 | BC005164 |
| H200014719 | ACTA2 | actin, alpha 2, smooth muscle, aorta | NM_001613 | X13839 |
| H200011577 | TMEM30A | transmembrane protein 30A | NM_018247 | AL832815 |
| H300021495 | NP_073604 | hypothetical protein FLJ12484 | NM_022767 | BC005164 |
| H300015667 | NP_073604 | hypothetical protein FLJ12484 | NM_022767 | BC005164 |
| H200016323 | NP_689426 | p53 target zinc finger protein | NM_152240 | AK122768,AK122768 |
| H300022025 | BAX | BCL2-associated X protein | NM_1387610 | - |
| H300000421 | 15E1_HUMAN | hypothetical protein HSPC132 | NM_016399 | - |
| H300020656 | NP_073604 | hypothetical protein FLJ12484 | NM_022767 | BC005164 |
| H200000088 | XPC | xeroderma pigmentosum,  complementation group C | NM_004628 | BC016620,D21089 |
| H200008365 | CDKN1A | cyclin-dependent kinase inhibitor 1A (p21, Cip1) | NM_0784670 | U03106,BC000312 |
| H300003103 | PHPT1 | phosphohistidine phosphatase 1 | NM_014172 | - |
| H300021798 | TP53I3 | tumor protein p53 inducible protein 3 | NM_1471840 | - |
| H300022581 | TP53I3 | tumor protein p53 inducible protein 3 | NM_1471840 | AF010309 |
| H200009234 | - | hypothetical protein MGC2941 | NM_024297 | - |
| H300020858 | HNRPDL | heterogeneous nuclear ribonucleoprotein D-like | NM_0313720 | D89678 |
| H200006609 | NP_060189 | SNF-1 related kinase | NM_017719 | D43636,CR749621 |
| H300008995 | PRKCH | protein kinase C, eta | NM_006255 | BC037268 |

Note: 25 probe model represents 18 genes (genes are represented by multiple probe IDs)
