# Supplementary material for: Gene Expression Signatures That Predict Radiation Exposure in Mice and Humans
Source: PLoS Med. 2007 Apr 3;4(4):e106. doi: 10.1371/journal.pmed.0040106 (PMC1845155; doi:10.1371/journal.pmed.0040106)
Supplement: Supplementary file 5 [file pmed.0040106.st005.doc]

| **GO Ontology of genes found in the  human radiation signature** | **Genes in list  with GO term** | **Genes within Human Genome with annotation in GO term** | **ln(Bayes factor)** | **Genes in list** |
| --- | --- | --- | --- | --- |
| induction of apoptosis by intracellular signals | 2 | 14 | 6.6 | CDKN1A TP53I3 |
| induction of programmed cell death | 3 | 120 | 5.96 | BAX CDKN1A TP53I3 |
| induction of apoptosis | 3 | 120 | 5.96 | BAX CDKN1A TP53I3 |
| nucleotide-excision repair | 2 | 21 | 5.86 | DDB2 XPC |
| positive regulation of apoptosis | 3 | 127 | 5.81 | BAX CDKN1A TP53I3 |
| positive regulation of programmed cell death | 3 | 128 | 5.78 | BAX CDKN1A TP53I3 |
| apoptosis | 4 | 436 | 4.86 | BAX BBC3 CDKN1A TP53I3 |
| programmed cell death | 4 | 438 | 4.84 | BAX BBC3 CDKN1A TP53I3 |
| cell death | 4 | 466 | 4.61 | BAX BBC3 CDKN1A TP53I3 |
| death | 4 | 470 | 4.57 | BAX BBC3 CDKN1A TP53I3 |
| regulation of apoptosis | 3 | 247 | 3.92 | BAX CDKN1A TP53I3 |
| regulation of programmed cell death | 3 | 249 | 3.89 | BAX CDKN1A TP53I3 |
| positive regulation of cellular physiological process | 3 | 258 | 3.8 | BAX CDKN1A TP53I3 |
| induction of apoptosis by oxidative stress | 1 | 1 | 3.68 | TP53I3 |
| positive regulation of physiological process | 3 | 334 | 3.09 | BAX CDKN1A TP53I3 |
| regulation of survival gene product activity | 1 | 3 | 2.98 | BAX |
| negative regulation of survival gene product activity | 1 | 3 | 2.98 | BAX |
| apoptotic mitochondrial changes | 1 | 5 | 2.58 | BAX |
| germ cell development | 1 | 6 | 2.43 | BAX |
| DNA repair | 2 | 180 | 1.83 | DDB2 XPC |
| regulation of cellular physiological process | 3 | 564 | 1.7 | BAX CDKN1A TP53I3 |
| response to DNA damage stimulus | 2 | 199 | 1.64 | DDB2 XPC |
| response to endogenous stimulus | 2 | 212 | 1.53 | DDB2 XPC |
| negative regulation of cellular physiological process | 2 | 263 | 1.14 | BAX CDKN1A |
| induction of apoptosis by extracellular signals | 1 | 28 | 1.03 | BAX |
